# Deletion of interleukin-6 in monocytes/macrophages suppresses the initiation of hepatocellular carcinoma in mice

**DOI:** 10.1186/s13046-016-0412-1

**Published:** 2016-09-02

**Authors:** Lingxiang Kong, Yongjie Zhou, Hong Bu, Tao Lv, Yujun Shi, Jiayin Yang

**Affiliations:** 1 Department of Hepato-Biliary-Pancreatic Surgery, West China Hospital of Sichuan University, Chengdu, 610041 China; 2 Laboratory of Pathology, West China Hospital, Sichuan University, Chengdu, 610041 China; 3 Key Laboratory of Transplant Engineering and Immunology, Ministry of Health, Chengdu, 610041 China

**Keywords:** Hepatocellular carcinoma (HCC), Inflammation, Tumor associated macrophage (TAM), Interleukin- 6 (IL-6)

## Abstract

**Background:**

Hepatocellular carcinoma (HCC) is associated with inflammation, and roughly 30 % of the global population shows serological evidence of current or past infection with hepatitis B or hepatitis C virus. Resident hepatic macrophages, known as Kupffer cells (KCs), are considered as the specific tumor-associated macrophages (TAMs) of HCC, and can produce various cytokines—most importantly interleukin (IL)-6—to promote tumorigenesis of HCC. However, the roles of KCs and IL-6 in carcinogenesis in the liver are still unclear.

**Methods:**

We analyzed leukocyte-related peripheral blood data of 192 patients and constructed a mouse model in which the bone marrow was cleared out by irradiation and reconstructed using bone marrow donated from IL-6-deficient mice to further elucidate the hepatic pathological changes in response to toxic challenge and oncogenic gene mutation.

**Results:**

Peripheral monocyte counts and serum IL-6 levels were significantly higher in patients with HCC than in those without HCC. In addition, there was a significant difference in the levels of IL-6 among individuals with different histopathological grades. In mice with selective IL-6 ablation in monocytes/KCs, we observed decreased toxic liver injury, inflammatory infiltration, and systemic inflammation. In Mdr2-deficient mice, which spontaneously developed HCC, the loss of IL-6 in monocytes/KCs resulted in inhibition of IL-6/signal transducer and activator of transcription 3 signaling, decreased serum IL-6 levels, and delayed tumorigenesis.

**Conclusions:**

Our findings demonstrate that increased TAM-derived IL-6 had an amplifying effect on the inflammation response, thereby promoting the occurrence and development of HCC.

## Background

Hepatocellular carcinoma (HCC) is the most common type of primary tumor of the liver, representing approximately 85–90 % of primary hepatic malignancies. HCC is ranked the fifth and seventh most common cancer in men and women, respectively, and represents the third leading cause of neoplasm-related death worldwide [1].

Since the first documented proposition of an association between inflammation and cancer by the German pathologist Rudolf Virchow Chronic in the mid-19th century, our understanding of inflammation and cancer has improved dramatically [2]. Immune activation and persistent inflammation have been shown to contribute to malignancy, and local chronic tissue inflammation often leads to malignant transformation [3, 4]. Furthermore, a randomized clinical trial showed that patients receiving long-term therapy (>5 years) with anti-inflammatory drugs, such as aspirin, had fewer relapses or appearances of new tumors, particularly colon cancer, providing further evidence for the pro-cancer effects of long-term inflammation [5]. HCC has been shown to be associated with chronic viral infection, such as hepatitis B virus (HBV) and hepatitis c virus (HCV) [6, 7], and is thought to be driven by inflammation. Moreover, roughly 30 % of the world’s population shows serological evidence of current or past HBV or HCV infection, with particularly high infection rates in China [8]; Thus, elucidation of the relationship between HCC and inflammation is critical for the development of improved therapies to manage HCC.

Monocytes are an important type of leukocytes and can differentiate into macrophages. Within the tumor microenvironment, macrophages can further differentiate into TAMs. TAMs are the main population of inflammatory cells in solid tumors and release cytokines that contribute to the occurrence and development of tumors [9]. Additionally, macrophages have both anti- and pro-cancer activities through the expression of different functional programs in response to distinct microenvironmental signals [10]. KCs, the resident hepatic macrophages, are considered HCC-specific TAMs. KCs make up less than 5 % of the volume of the liver; however, they represent about 80 % of the total fixed macrophage population in the human body [11]. Owing to their wide range of biological functions in immune regulation, inflammation, and oncogenesis, KCs are involved in the pathogenesis of several liver diseases. As a basic protective function and the first line of defense in the healthy liver, KCs exert immune functions through phagocytosis and antigen presentation for components entering from the portal vein and arterial circulation. However, the initial activation of KCs can cause liver injury and hepatocyte cell death through the release of inflammatory cytokines and production of matrix metalloproteinases (MMPs), thereby contributing to later stages of wound healing and fibrosis regression and eventually leading to liver cirrhosis [12]. The development of HCC occurs in about one-third of individuals with cirrhosis and is closely related to KCs. Based on phenotypic polarization, the state of activation of KCs can be classified as M_1_ (classically activated) or M_2_ (alternatively activated) [13]. Within the tumor microenvironment, TAMs are mainly polarized towards the M_2_ phenotype [14]. Although the specific mechanism is still unclear, macrophage polarization is strongly related to tumor stage, suggesting that dynamic switching from the M_1_ phenotype during the early phases of chronic inflammation to an M_2_-like phenotype may occur in established tumors [15].

IL-6 is a pleiotropic cytokine that is mainly produced by macrophages and lymphocytes [16]. IL-6 is one of the key factors involved in the inflammatory response associated with the course of hepatitis due to hepatitis virus-related infection [17, 18]. In the inflammatory response, IL-6 acts as an activator by controlling the hepatic acute phase (AP) response [19], which is critical for the maintenance of body homeostasis during infections and inflammatory conditions after trauma, burns, tissue necrosis, and surgery [20]. IL-6 can activate several pathways, including the Janus kinase (JAK)/signal transducer and activator of transcription (STAT), p38 mitogen-activated protein kinase (MAPK), extracellular signal-regulated kinase (ERK), and phosphoinositol 3-kinase (PI3K) pathways, ultimately leading to cell proliferation, protection from apoptosis, and increased metastatic potential [18]. Of these signaling pathways, the JAK/STAT pathway is one of the most important in mediating the inflammatory response. In this pathway, IL-6 family members interact with a receptor complex on the cell surface. IL-6 first binds to the IL-6 receptor (glycoprotein (gp) 80) and then interacts with gp130. Subsequently, dimerization of two gp130 molecules activates JAKs, which phosphorylate specific tyrosine residues of gp130 and thus activate the transcription factors STAT3 and other potential pathways, resulting in the activation of the AP response [21]. STAT3 was initially described as a DNA-binding factor expressed in TAM-derived IL-6-stimulated hepatocytes and has been shown to be capable of selectively interacting with an enhancer element in the promoter of AP genes, known as AP response elements [22]. In rodents, IL-6 is a progrowth factor required for hepatic regeneration and survival after partial hepatectomy [23, 24]. In mice, Streetz et al. suggested that IL-6 has a protective role in non-parenchymal liver cells during fibrosis progression in the pathogenesis of chronic liver diseases [25]. Moreover, in humans, Wieckowska et al. reported that IL-6 expression is markedly higher in the livers of patients with nonalcoholic steatohepatitis (NASH) than in patients with simple steatosis or normal biopsies [26]. Additionally, IL-6 expression in hepatocytes is positively correlated with the degree of inflammation and stage of fibrosis in these patients [26]. Many clinical studies have also shown that IL-6 expression is increased in HCC [27, 28]. Taken together, these findings support that the role of IL-6 in liver disease varies greatly between humans and animals, and the direct relationships between IL-6 and HCC are still unclear.

Although many studies have demonstrated that HCC is closely correlated with aberrant IL-6 expression, the direct relationships between TAM-derived IL-6 and HCC are still not clear. In order to explore the role of TAM-derived IL-6 in the occurrence and development of HCC, the most important initial step is to establish a suitable model. Currently, there are three models that may be consistent with our experimental objective; these include gadolinium chloride (GdCl_3_)-induced, systemic IL-6-knockout, and conditional gp130-knockout (Cre/loxP system) murine models. However, all of these models are associated with large biases, which may result in unreliable results.

Therefore, to investigate the central role of TAM-derived IL-6 in the occurrence and development of HCC, we developed a monocyte-selective IL-6-deficient mouse model using mice in which the bone marrow was deconstructed and then reconstituted with bone marrow donated from a systemic IL-6-deficient mouse. We elucidated the occurrence and development of liver cancer in response to toxin challenge and *mdr2* gene ablation in the monocyte-specific IL-6-deficient mice.

## Methods

### Patients

A total of 192 patients (HCC = 113 and Non-HCC = 59) from Jun.1, 2015 to Feb.1 2016 in our center were included in this study. The Non-HCC group consists of the donors of living donor liver transplantation (*n* = 10), patients with hepatic hemangioma (*n* = 19) or chronic liver disease (*n* = 30). All of the data of HCC patients come from the patients who are undergoing liver resection (HR) or liver transplantation (LT), and diagnosis was confirmed by histopathologic examination of the surgical samples. To ensure the consistency of baseline data, all patients are diagnosed with HCC for the first time, and with non-liver infectious diseases such as urinary, respiratory or other system inflammation.

### Mice and treatment

(B6 × 129) F2 (B6.129) mice homozygous for *IL-6* mutation was donated by Dr. Yu, West China Hospital, Sichuan University. We chose the *mdr2*^*−/−*^ mice as a spontaneous tumor model which highly mimics the inflammation-associated HCC [29]. The mice were maintained on an alternating 12-h light/dark cycle, fed regular chow, and given water ad libitum.

### Bone marrow transplantation

Four to ten weeks old *IL-6*^*−/−*^ or littermate mice were used as bone marrow (BM) donors and the BM cells were isolated from their femur, tibia, and humerus. Ten-week old recipient Balb/c mice fed with medicated water one week before irradiation. To ablation the BM, mice were exposed to a dose of 8 Gy γ-irradiation. Then the mice were transplanted with 10^6^ BM cells via tail vein within six hours following ion exposure. The experimental design is illustrated in Fig. 2a.

### Histological staining

Liver tissues were fixed in 10 % formaldehyde and the 4-μm sections were subjected to hematoxylin and eosin staining. For immunohistochemistry staining, paraffin-embedded liver sections were incubated with appropriately diluted antibodies. The results were developed by the 3,3’-diaminobenzidine (DAB) method.

### Mouse monocyte isolation

Mouse blood was collected and added into EDTA vacutainer tubes. Erythrocytes were lysed using red blood cell (RBC) lysis solution for 5 min at room temperature. Cells were centrifuged at 1000 g for 3 min to remove RBC lysis solution, and the leukocyte pellet was resuspended and washed with phosphate-buffered saline (PBS). Cell viability was counted by trypan blue exclusion.

### mRNA isolation and real-time RT-PCR

Total mRNA was purified from isolated circulating monocytes with Trizolreagent (Invitrogen, United States). mRNA was reverse transcribed to cDNA using an iScript cDNA Synthesis kit (Bio-Rad, United States). Q-PCR reactions were performed using a Bio-Rad CFX96TM Real-Time PCR system with SsoFastTM EvaGreen Supermix (Bio-Rad). The relative expression of target genes was normalized to an internal β-actin control. Relative gene expression with a greater than 2-fold change was considered statistically significant. The sequences of mouse IL-6 gene primers were: upstream, 5’-CAC GGC CTT CCC TAC TTC ACATG AGG ACA CTT GCC TGG TG-3’, and downstream, 5’-TTC TGC AAG TGC ATC ATC GT-3’.

### Cytokine level analysis

Serum cytokine levels were analyzed on a Luminex 200 system using a Millipore Map Mouse Cytokine/Chemokine Magnetic Bead Panel kit (Millipore, United States) according to the manufacturer’s instructions.

### Western blot analysis

The livers were homogenized for protein extraction. Sodium dodecyl sulfate polyacrylamide gel electrophoresis (SDS-PAGE) and immunoblotting were performed and an ECL reagent was used for chemiluminescence detection.

### Statistical analysis

All results were expressed as the mean ± SD. SPSS 18.0 statistical software (SPSS Inc., Chicago, IL, USA) and GraphPad Prism 5 software were used to analyse the relevant data. For statistical analysis, Categorical data were presented as number (per cent) and compared using Pearson chi-Square, Fisher’s exact test. Continuous variables were expressed as the mean value ± SD and analysed using *t*-test. *P* < 0.05 was considered statistically significant.

## Results

### Clinical data for patients with and without HCC

**Fig. 1:**
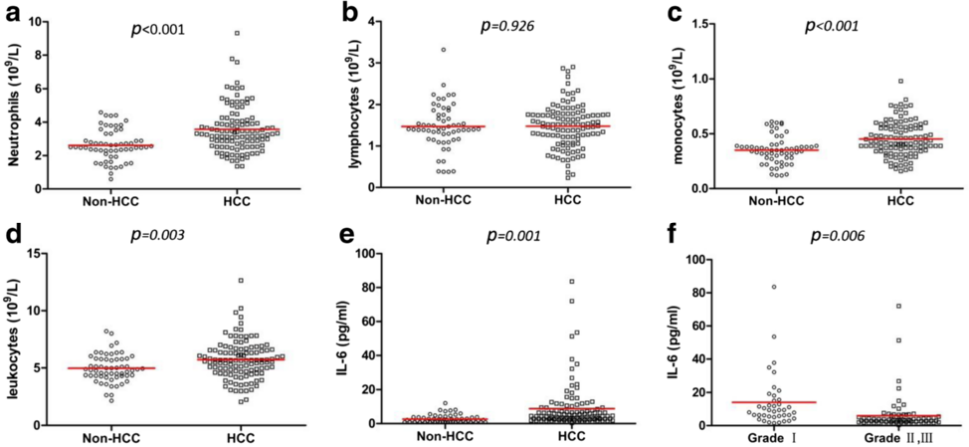
Peripheral blood data for patients. **a**-**e** The counts of neutrophils, lymphocytes, monocytes, total leukocytes in the blood and serum levels of IL-6 in the HCC (*n* = 113) and Non-HCC group (*n* = 59) (3.57 ± 1.38 vs. 2.62 ± 0.93, 10^9^/L, *P* < 0.001; 1.48 ± 0.52 vs. 1.47 ± 0.54, 10^9^/L, *P* = 0.926; 0.45 ± 0.16 vs. 0.35 ± 0.13, 10^9^/L, *P* < 0.001; 5.71 ± 1.67 vs. 4.96 ± 1.20, 10^9^/L, *P* = 0.003; 8.75 ± 13.13 vs. 2.66 ± 2.36, pg/ml, *P* = 0.001 respectively) (**f**) Serum IL-6 level in the histopathologic gradesIgroup (*n* = 39) and gradesII, III group (*n* = 74) (14.02 ± 15.95 vs. 5.98 ± 10.46, pg/ml, *P* = 0.006)

In our clinical retrospective observation, a total of 155 cases were selected (with HCC, *n* = 113; without HCC, *n* = 59). The baseline characteristics and disease features of patients with and without HCC and patients with HCC classified as histopathological grades I, II, and III were summarized in Table 1. There were no significant differences between patients with and without HCC and among patients with grades I, II, or III HCC in terms of baseline demographics and disease features. Moreover, there were no significant differences in the numbers of lymphocytes in patients with or without HCC (1.48 ± 0.52 × 10^9^/L versus 1.47 ± 0.54 × 10^9^/L, respectively; *P* = 0.926; Fig. 1b). In contrast, the counts of peripheral neutrophils, monocytes, leukocytes, and serum IL-6 levels were significant higher in patients with HCC than in patients without HCC (Fig. 1a, c-e). In addition, there was a significant difference in IL-6 expression among individuals with different histopathological grades (Fig. 1f).

**Table 1:** Baseline demographic and disease features characteristics

Variables	HCC	Non-HCC	*P* value	Grades I	Grades II, III	*P* value
	(*n* = 113)	(*n* = 59)		(*n* = 39)	(*n* = 74)	
Age (mean ± SD,years)	53.66 ± 11.52	51.52 ± 11.63	0.250	55.44 ± 12.28	53.37 ± 11.81	0.405
Male (%)	90 (79.6 %)	44 (74.6 %)	0.447	29 (80.6 %)	55 (82.1 %)	0.848
Height (mean ± SD, cm)	164.64 ± 6.36	164.80 ± 3.80	0.833	163.25 ± 7.11	165.33 ± 6.34	0.131
Weight (mean ± SD, kg)	63.17 ± 10.33	61.69 ± 5.68	0.227	62.38 ± 10.22	63.77 ± 11.18	0.539
BMI (mean ± SD, kg/m^2^)	23.25 ± 3.17	22.68 ± 1.60	0.123	23.36 ± 3.25	23.25 ± 3.37	0.875
HBV positivity (%)	82 (72.6 %)	38 (64.4 %)	0.269	26 (72.2 %)	50 (74.6)	0.791
Number of tumors (>1,%)	-	-	-	9 (23.1 %)	18 (24.3 %)	0.882
Largest tumor size (mean ± SD, cm)	-	-	-	4.83 ± 2.77	4.68 ± 1.98	0.743

*BMI* Body mass index, *HBV* hepatitis B virus

### Construction of monocyte-specific IL-6-deficient mice

**Fig. 2:**
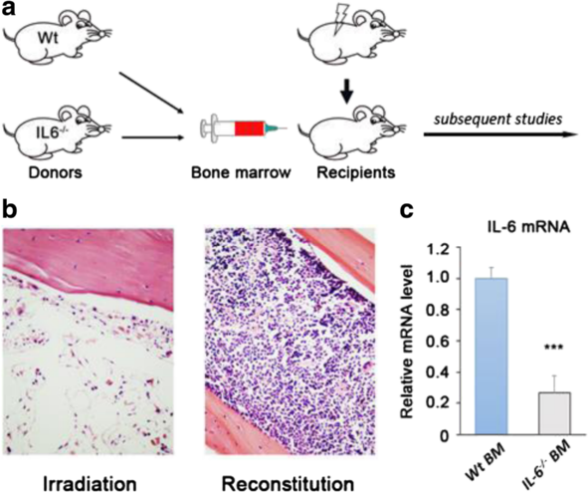
Reconstitution of the bone marrow with IL-6 deficient donors. **a** Schematic representation of the experimental design. **b** Histology examination of the deconstruction and reconstruction of the bone marrow. **c** The transcription levels of IL-6 mRNA in circulating monocytes. Each bar represents the mean ± s.e.m from ≥3 independent quantitative PCR experiments. Gaphd was used as loading control.***p < 0.001

Ion irradiation at 8 Gy completely cleared out the BM in all recipient mice (Fig. 2b), and mice died within 7 days if BM transplantation was not performed. BM was reconstituted after the infusion of BM donated by either *IL-6*^*−/−*^ or *WT* donors (Fig. 2b). Mice that survived for more than 1 month after BM transplantation were considered to have undergone successful BM reconstitution and were used for further experiments.

To confirm IL-6 disruption in monocytes, we activated circulating monocytes by intraperitoneal injection of lipopolysaccharide (LPS) at 1 μg/kg bodyweight (BW) and isolated monocytes from the blood 60 min later. We performed quantitative polymerase chain reaction (PCR) to quantify *IL-6* mRNA transcription in circulating monocytes. The results showed that *IL-6* mRNA levels in the mice receiving IL-6 deficient BM were substantially decreased (Fig. 2c).

### IL-6 deprivation in monocytes decreased toxic liver injury

**Fig. 3:**
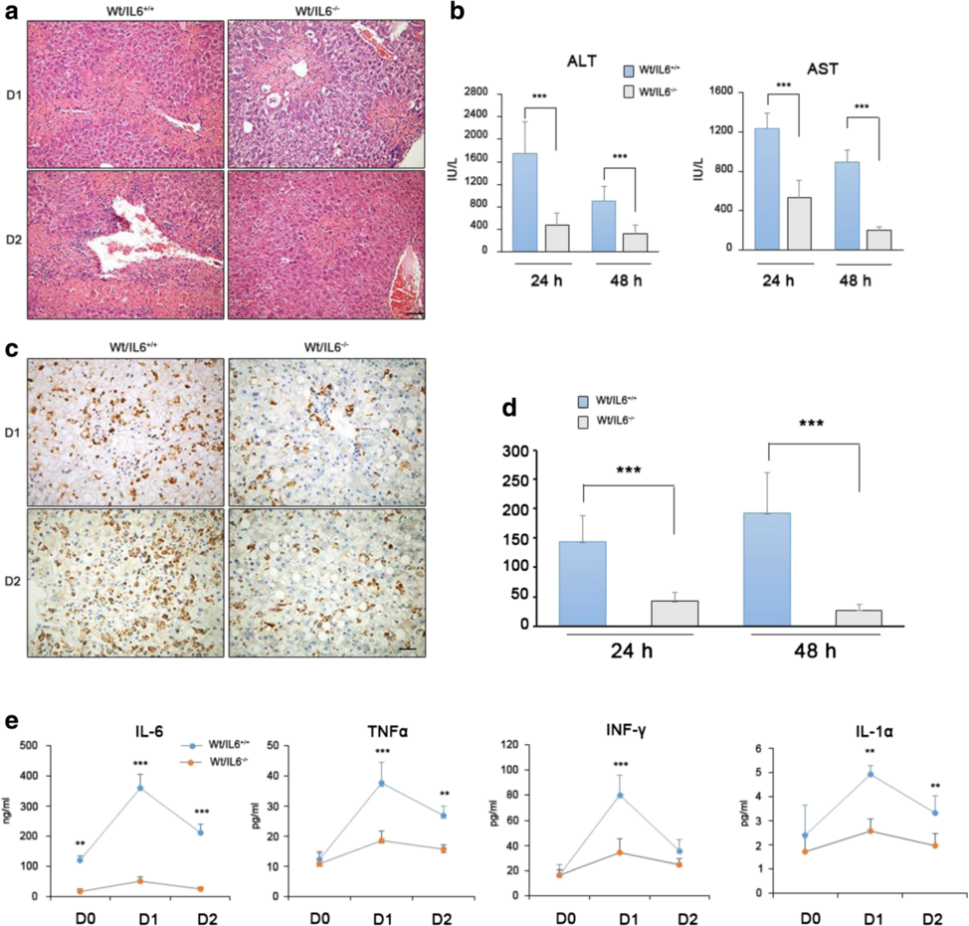
IL-6 deprivation in monocytes decreases toxic liver injury. **a**-**b** Specific loss of IL-6 in monocytes improves liver histology and decreases toxic liver injury. **c**-**d** Less macrophage aggregation in liver tissue when their IL-6 was deprived. The numbers of F4/80-positive cells were counted in 10 consecutive high-power fields. Each bar represents the mean ± s.e.m of positive cells in liver sections from three mice. ***p < 0.001. **e** IL-6 ablation in monocytes suppresses circulating inflammatory factors. Data are presented as mean ± s.e.m, *n* ≥ 6; **p < 0.01 and ***p < 0.001 between two groups, respectively. Scale bar = 100 μm

Hepatic macrophages, derived either from the renewal of resident KCs or from recruitment of circulating monocytes, play critical roles in initiation and exacerbation of acute liver damage in response to toxin challenge [12]. Thus, we next treated WT mice with irradiation and reconstituted the BM by donation of BM from IL-6-deficient or WT mice (described as *Wt/IL6*^*−/−*^ and *Wt/IL6*^*+/+*^ mice, respectively). After successful BM reconstitution, the mice were intraperitoneally injected with 10 % carbon tetrachloride (CCl_4_) solution in olive oil at 10 mL/kg body weight (BW). Liver specimens and blood samples were harvested on days 1 and 2 after toxin injection. *Wt/IL6*^*+/+*^ mice showed severe liver damage and their hepatic indexes, including alanine aminotransferase (ALT) and glutamic-oxalacetic transaminase (AST), were dramatically increased on day 1, with further deterioration observed on day 2. In contrast, in *Wt/IL6*^*−*/*−*^ mice, liver histology was well maintained, and hepatic indexes were mildly to moderately increased (Fig. 3a, b).

Next, we examined the infiltration of macrophages in the liver. As illustrated by F4/80 immunoreactivity, in *WT/IL6*^*+/+*^ mice, macrophages were aggregated around the necrotic parenchyma, whereas fewer F4/80-positive cells were observed in *WT/IL6*^*−/−*^ mice (Fig. 3c, d).

Acute hepatic damage is often accompanied with a systemic inflammatory response. Therefore, we examined the levels of circulating factors, including IL-6, tumor necrosis factor (TNF)-α, interferon (INF)-γ, and IL-1α. As expected, all of these factors were significantly upregulated in *WT/IL6*^*+/+*^ mice as compared with their levels in *WT/IL6*^*−/−*^ mice (Fig. 3e).

### Inflammatory infiltration and systemic inflammation were reduced in IL-6-deficient mice

**Fig. 4:**
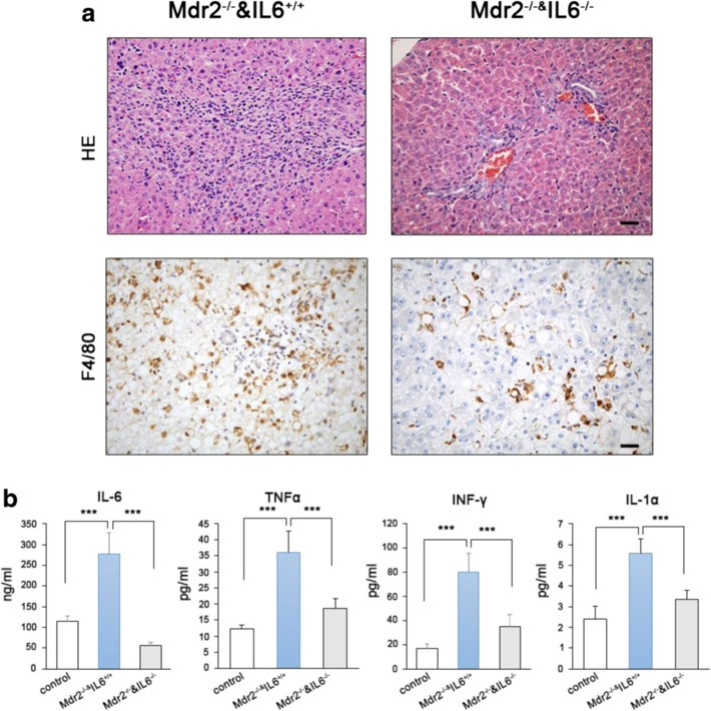
Loss of IL-6 in monocytes decreases spontaneous liver damage in mdr2^−/−^ mice. **a** Loss of IL-6 in monocytes improves hepatic histology and inhibits the infiltration of macrophages (scale bar = 50 μm). **b** IL-6 ablation in monocytes suppresses circulating inflammatory factors in mdr2^−/−^ mice. Data are presented as mean ± s.e.m, *n* ≥ 6; ***p < 0.001

*Mdr2*-deficient mice lack the ability to secrete phospholipid into the bile from hepatocytes and develop spontaneous chronic non-suppurative inflammatory cholangitis with portal inflammation and ductular proliferation [29]. We next replaced the BM of *Mdr2*^*−/−*^ mice with IL-6-deficient BM (described as *Mdr2*^*−/−*^*/IL6*^*−/−*^ and *Mdr2*^*−/−*^*/IL6*^*+/+*^ mice, respectively) and then examined inflammatory infiltration in the liver at 2 months of age. In *Mdr2*^*−/−*^*IL6*^*+/+*^ mice, liver histology was noticeably impaired, and obvious inflammatory cell infiltration was observed in the parenchyma (Fig. 4a). Although hepatocyte injury was also prominent in *Mdr2*^*−/−*^*/IL-6*^*−/−*^ mice, the numbers of infiltrated inflammatory cells and F4/80-positive cells were greatly lower in livers from these mice than in livers from *Mdr2*^*−/−*^*/IL6*^*+/+*^ mice (Fig. 4a).

We then measured the levels of circulating cytokines in mice of both genotypes. As expected, all cytokines examined in this study were substantially increased in *Mdr2*^*−/−*^*IL6*^*+/+*^ mice and moderately elevated in *Mdr2*^*−/−*^*IL6*^*−/−*^ mice as compared with those in WT mice (Fig. 4b).

### IL-6-deficient mice exhibited a lower rate of spontaneous liver cancer than WT mice

**Fig. 5:**
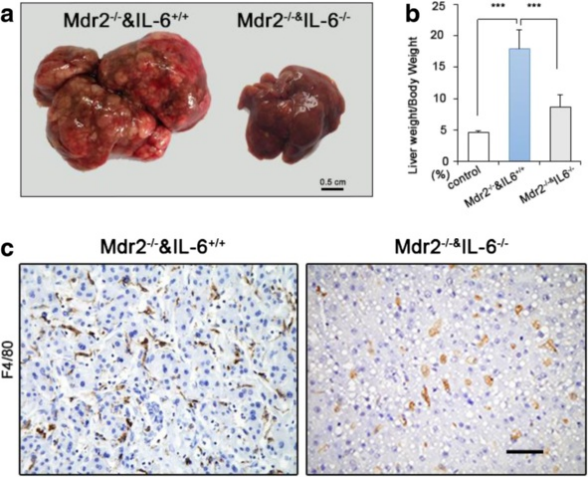
Loss of IL-6 in monocytes decreases spontaneous liver cancer in mdr2^−/−^ mice. **a** The gross view of spontaneous liver cancer in mice. **b** The mean ratio of the LW/BW between mice with different bone marrow transplantation. Data are presented as mean ± s.e.m, *n* ≥ 6; ***p < 0.001. **c** Greatly less infiltrated macrophages in tumor tissue of *Mdr2* ^*−/−*^ & *IL6* ^*−/−*^mice. Scale bar = 50 μm

Because *Mdr2* ablation leads to early spontaneous carcinogenesis at 4–6 months of age [29], we examined the development of liver cancer at 6 months of age in IL-6-deficient and WT mice. All 12 *Mdr2*^*−/−*^*IL-6*^*+/+*^ mice and four of 13 *Mdr2*^*−/−*^*IL6*^*−/−*^ mice developed liver cancer. Moreover, the mean tumor size was much smaller in *Mdr2*^*−/−*^*IL6*^*−/−*^ mice than in *Mdr2*^*−/−*^*IL6*^*+/+*^ mice (Fig. 5a). A comparison of the ratio of liver weight (LW) to BW showed that LW/BW was much lower in *Mdr2*^*−/−*^*IL6*^*−/−*^ mice than in *Mdr2*^*−/−*^*IL-6*^*+/+*^ mice (Fig. 5b).

Next, we examined the expansion of macrophages in the liver. The infiltration of F4/80-positive cells was rare in *Mdr2*^*−/−*^*IL6*^*−/−*^ mice. However, noticeable aggregation of macrophages was observed in *Mdr2*^*−/−*^*IL-6*^*+/+*^ mice (Fig. 5c).

### IL-6 was upregulated in the tumors of Mdr2^−/−^IL-6^+/+^ mice

**Fig. 6:**
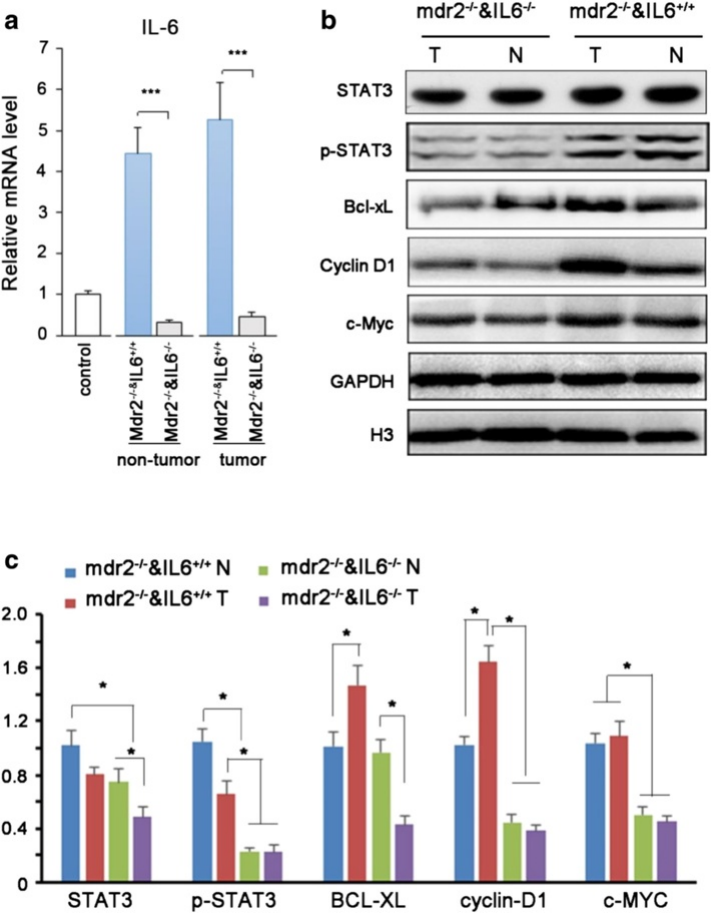
Loss of IL-6 in monocytes suppresses tumor growth signaling in mdr2^−/−^ mice. **a** The transcription levels of IL-6 mRNA in tumor and non-tumor tissues. **b**-**c** Immunoblotting of proteins involved in IL-6 signaling pathway. GAPDH and histone H3 were used as loading control of cytoplasmic protein and nuclear protein, respectively. The levels of the indicated proteins are expressed as ratios to their loading control. Each bar represents the mean ± s.e.m from ≥3 independent experiments. *p < 0.05

We speculated that high concentrations of IL-6 secreted by local macrophages were associated with a high rate of tumorigenesis. Therefore, we next examined the levels of IL-6 in liver homogenates. As expected, IL-6 was significantly decreased in both the surrounding tissues and tumor tissues in *Mdr2*^*−/−*^*IL6*^*−/−*^ mice (Fig. 6a).

STAT3 is a target of IL-6. After stimulation by IL-6, STAT3 is phosphorylated, translocates to the cell nucleus, and then functions as a transcriptional activator to regulate various genes in response to cell stimuli, thereby playing key roles in many cellular processes, such as cell growth and apoptosis. The IL-6/STAT3 cascade plays a key role in the promotion of hepatocyte proliferation, liver repair, and tumorigenesis by protecting cells from apoptotic stimuli and enhancing cell-cycle progression. Therefore, we next investigated the involvement of STAT3 in the transduction of IL-6 signal cascades. Immunoblotting for phospho-STAT3 showed that phospho-STAT3 was down-regulated in *Mdr2*^*−/−*^*IL6*^*−/−*^ mice. At the same time, proteins downstream of STAT3, including Bcl-XL, cyclin D1, and c-Myc, were robustly increased in *Mdr2*^*−/−*^*IL6*^*+/+*^ mice and moderately increased in *Mdr2*^*−/−*^*IL6*^*−/−*^ mice (Fig. 6b, c).

## Discussion

In this study, we found that IL-6 levels and monocyte numbers were significantly higher in peripheral blood samples from patients with HCC than in patients without HCC. Additionally, IL-6 expression was related to the tumor grade in patients with HCC and had moderate predictive value in relation to HCC. In the monocyte-selective IL-6-deficient mouse model, we found that *WT/IL6*^*+/+*^ mice induced by CCl_4_ exhibited higher levels of toxic liver injury, inflammatory infiltration, and systemic inflammation. Using a spontaneous tumorigenesis model that closely imitated inflammatory tumors, we found that *Mdr2*^*−/−*^*IL6*^*+/+*^ mice had higher IL-6 levels in tumors and higher rates of early spontaneous liver cancer. Furthermore, analysis of the molecular mechanisms showed the activation of STAT3 and its downstream anti-apoptotic and pro-proliferation genes in the *Mdr2*^*−/−*^*IL6*^*+/+*^ mice.

The inflammatory reaction is a complex double-edged biological response in which leukocytes are critical and the number of leukocytes often reflects the severity of inflammation in patients. Based on the peripheral blood data in our study, we determined that patients with HCC had higher leukocyte counts than patients without HCC, consistent with a report by Jian-Shong et al. [30]. In complete blood cell counts, leukocytes mainly include neutrophils (≈62 %), lymphocytes (≈30 %), and monocytes (≈5.3 %). Normally, IL-6 is not secreted by neutrophils, but promotes the release of neutrophils from BM. Although IL-6 is mainly produced by macrophages and lymphocytes, lymphocytes did not show any significant differences between patients with or without HCC. In addition, in humans, about 80 % of the total fixed macrophages in the body are located in the liver. Taken together, these findings together and our current findings suggested that the occurrence and development of HCC may be related to the level of IL-6 secreted by macrophages and that a high level of IL-6 in turn promotes an increase in the number of leukocytes. However, the specific mechanisms mediating these effects are still not clear. Accordingly, based on the central role played by IL-6 in the occurrence and development of HCC, we developed an appropriate mouse model to comprehensively elucidate the gradual pathological changes in the liver that occurred over time.

In previous studies, in order to decrease IL-6 levels in the liver, researchers often used GdCl_3_ to deplete macrophages. However, many studies have demonstrated that GdCl_3_, as a specific activator of calcium-sensing receptors (CaSRs), does not just deplete KCs but also has a wide range of other effects [31]. GdCl_3_ can suppress a broad range of macrophage-related cell markers and cytokines, including TNF-α, IL-6, and IL-1B [32, 33]. At the same time, GdCl_3_ can reduce the levels of peroxisomal protein (PXP), CYP311, cytochrome P450, and many functional mRNAs [34–37]. Systemic knockout of IL-6 in mice has improved our understanding of the role of IL-6 in hepatic pathophysiology to a greater degree than GdCl_3_. However, systemic loss of IL-6 often leads to spontaneous liver impairment and damaged regeneration [38]. Therefore, we cannot investigate the role of IL-6 in the development of liver cancer using this transgenic model. Gp130 as a part of a receptor complex on the cell surface, is crucial for all IL-6 family members because they use this receptor for signal transduction [25]. Although conditional knockout of gp130 using a Cre/LoxP system successfully prevents embryonic lethality and specifically targets in nonparenchymal liver cells or hepatocytes, gp130 knockout is not sufficient to explain the role of monocytes in the pathogenesis of liver diseases. Therefore, to overcome these challenges, we constructed a mouse model in which the BM was depleted by irradiation and then reconstituted using BM from an IL-6-deficient mouse donor. In this model, IL-6 was disrupted in all nucleated cells derived from the BM, including monocytes, dendritic cells, neutrophils, and macrophages.

IL-6 is an essential activator for controlling the hepatic AP response. Kovalovich et al. found that IL-6^*−/−*^ mice develop more severe liver fibrosis after CCl_4_ treatment [38]. In contrast, the *WT/IL6*^*+/+*^ mice induced by CCl_4_ treatment in our study showed severe liver damage, whereas liver histology in *WT/IL6*^*−/−*^ mice was fairly well maintained, with moderately increased hepatic indexes. One possible explanation of these observations is that monocyte-selective IL-6-deficient mice may be less influenced by other factors compared with traditional IL-6^*−/−*^ mice and may result in more targeted outcomes. Based on the above study, TAM-derived IL-6 can expand the inflammatory response, leading to liver damage rather than protective effects. IL-6 level is also consistently increased in patients with chronic inflammatory response [39, 40] and this is consistent with our clinical observations (Fig. 1e). Coincidently, HCC is thought to be driven by inflammation. Many experiments in mice have shown that nonspecific significant inhibition of hepatocyte proliferation in IL-6-deficient mice and the lack of IL-6 contributes to the increase in mortality [23]. Moreover, IL-6 treatment may be a therapeutic option for management of liver damage by promoting hepatocyte proliferation [24]. Interestingly, the outcomes in our study were not completely consistent with these previously established protective effects. In order to mimic inflammatory tumors, we chose *Mdr2*^*−/−*^ mice as a spontaneous tumorigenesis model [41]. In our study, we found that *Mdr2*^*−/−*^*IL-6*^*+/+*^ mice had a higher rate of spontaneous liver cancer and larger tumors. Therefore, we concluded that TAM-derived IL-6 may contribute to the occurrence and development of HCC rather than only promoting hepatocyte proliferation.

Molecule-targeted therapy as a new approach based on the study of HCC carcinogenic mechanisms may lead to the improvement of treatment options for patients with advanced HCC. After the development of the 2011 Barcelona Clinic Liver Cancer (BCLC) staging system, only one drug (the multikinase inhibitor sorafenib) has been approved for patients diagnosed with BCLC stage C HCC (accounted for 40 % of patients with HCC) [42]. Although molecular-targeted therapy is very important for moderate to advanced HCC, few effective drugs have been developed. Therefore, novel therapies are urgently needed. IL-6 has a key role in carcinogen-driven liver cancer development, promotes cancer cell proliferation, and inhibits the apoptosis of cancer cells through the activation of STAT3 [43–45, 17]. STAT3 activation induced by IL-6 via JAK has been implicated in colitis-associated colon cancer [46]. In our study, we focused on the IL-6/JAK/STAT3 pathway in the carcinogenesis of HCC. The levels of phospho-STAT3 as a target of IL-6 were dramatically decreased in *Mdr2*^*−/−*^*IL6*^*−/−*^ mice, and proteins downstream of STAT3, including Bcl-XL, cyclin D1, and c-Myc, were robustly increased in *Mdr2*^*−/−*^*IL6*^*+/+*^ mice. These findings suggested that IL-6 may promote tumor development by activating STAT3 and subsequently activating downstream STAT3 target genes, including the anti-apoptotic gene encoding Bcl-XL and the cell cycle promoting genes encoding cyclin-D1 and c-Myc. Our findings regarding the molecular mechanisms of IL-6 are consistent with above reports and may provide stronger evidence for the development of molecular-targeted therapy.

## Conclusions

In summary, TAM-derived IL-6 was highly correlated with the occurrence and development of HCC. Using a monocyte-selective IL-6-deficient mouse model of spontaneous inflammatory tumorigenesis, our findings directly demonstrated that the increased TAM-derived IL-6 had an amplifying effect on the inflammation response, and further promoted the occurrence and development of HCC. IL-6 played a vital role in carcinogenesis and this deserves the attention from physicians. This information may facilitate the development of novel strategies for the early prevention and treatment for patients with HCC by targeting the IL-6 system.
